# Comparative distribution of human and avian type sialic acid influenza receptors in the pig

**DOI:** 10.1186/1746-6148-6-4

**Published:** 2010-01-27

**Authors:** Rahul K Nelli, Suresh V Kuchipudi, Gavin A White, Belinda Baquero Perez, Stephen P Dunham, Kin-Chow Chang

**Affiliations:** 1 School of Veterinary Medicine and Science, University of Nottingham, Sutton Bonington Campus, College Road, Loughborough, Leicestershire LE12 5RD, UK; 2 Institute of Comparative Medicine, Faculty of Veterinary Medicine, University of Glasgow, Bearsden, Glasgow, G61 1QH, UK

## Abstract

**Background:**

A major determinant of influenza infection is the presence of virus receptors on susceptible host cells to which the viral haemagglutinin is able to bind. Avian viruses preferentially bind to sialic acid α2,3-galactose (SAα2,3-Gal) linked receptors, whereas human strains bind to sialic acid α2,6-galactose (SAα2,6-Gal) linked receptors. To date, there has been no detailed account published on the distribution of SA receptors in the pig, a model host that is susceptible to avian and human influenza subtypes, thus with potential for virus reassortment. We examined the relative expression and spatial distribution of SAα2,3-GalG(1-3)GalNAc and SAα2,6-Gal receptors in the major organs from normal post-weaned pigs by binding with lectins *Maackia amurensis agglutinins *(MAA II) and *Sambucus nigra agglutinin *(SNA) respectively.

**Results:**

Both SAα2,3-Gal and SAα2,6-Gal receptors were extensively detected in the major porcine organs examined (trachea, lung, liver, kidney, spleen, heart, skeletal muscle, cerebrum, small intestine and colon). Furthermore, distribution of both SA receptors in the pig respiratory tract closely resembled the published data of the human tract. Similar expression patterns of SA receptors between pig and human in other major organs were found, with exception of the intestinal tract. Unlike the limited reports on the scarcity of influenza receptors in human intestines, we found increasing presence of SAα2,3-Gal and SAα2,6-Gal receptors from duodenum to colon in the pig.

**Conclusions:**

The extensive presence of SAα2,3-Gal and SAα2,6-Gal receptors in the major organs examined suggests that each major organ may be permissive to influenza virus entry or infection. The high similarity of SA expression patterns between pig and human, in particular in the respiratory tract, suggests that pigs are not more likely to be potential hosts for virus reassortment than humans. Our finding of relative abundance of SA receptors in the pig intestines highlights a need for clarification on the presence of SA receptors in the human intestinal tract.

## Background

Influenza A viruses have a wide host range for birds and mammals, posing a major threat to animal health as well as a zoonotic threat to humans [1]. Influenza pandemics can arise from genetic reassortment between avian and human influenza viruses or alternatively by the direct adaptation of avian or mammalian viruses to efficient human to human transmission [2]. Swine influenza is a major respiratory problem in pigs; in uncomplicated infections the condition is usually mild to moderate and non-fatal, with complete recovery within 2 weeks after the onset of clinical signs. The 2009 pandemic H1N1 virus in experimentally infected pigs has been shown to produce similarly mild to moderate signs and pathology [3] as in most human cases of the same virus. The pig is often described as a mixing vessel for the reassortment of influenza viruses from different host species [4,5]. Indeed, the 2009 H1N1 pandemic virus has been shown to have originated from viruses of pig, avian and human origin [6]. A major determinant of influenza infection is the presence of virus receptors on susceptible host cells to which the viral haemagglutinin is able to bind. Avian influenza A viruses preferentially bind to sialic acid α2,3-galactose (SAα2,3-Gal) linked receptors, whereas human strains bind to sialic acid α2,6-galactose (SAα2,6-Gal) linked receptors [7-9]. As the porcine respiratory tract is the main predilection site for influenza infection and the porcine trachea possesses both SA receptors [10], the pig appears well placed to act as a vehicle for virus reassortment.

Although the pig is an important host species of influenza virus infection and in the evolution of the virus to cross species barrier, there is still no detailed information on the expression of its SA receptors. Knowledge of the distribution of SA receptors in the pig could facilitate our understanding of the pathogenesis and pathogenicity of the virus in the host. Although conventional swine and human influenza viruses are usually not life threatening in their respective host, the outcomes of highly pathogenic avian H5N1 infections in humans and pigs are very different. The mortality rate of human cases of H5N1 infections is in excess of 60% (257 deaths out of 417 official WHO cases) whereas the clinical effects of H5N1 in experimentally infected pigs are mild [11,12]. A comparative characterisation of the expression and distribution of SA receptors between pig and human may also provide an insight into differences in host response to the same virus. We report here on the relative expression and spatial distribution of SAα2,3-Gal and SAα2,6-Gal linked receptors in the major pig organs and make qualitative and functional comparisons with corresponding human tissues.

## Results

The lectins used were *Sambucus nigra *agglutinin (SNA) which is specific for SAα2,6-Gal (Shibuya et al., 1987), *Maackia amurensis *I (MAA I) and *Maackia amurensis *agglutinins (MAA II). The latter two are specific for SAα2,3-Galβ(1-4)GlcNAc and SAα2,3-Galβ(1-3)GalNAc respectively [13]. Overall, both human influenza (SA α2,6-Gal) and avian influenza (SA α2,3-Gal, MAA II specific) receptor types were extensively detected in the major pig organs examined. Each tissue showed distinctive spatial distribution of the two receptors.

### SA receptors distribution in respiratory tract

**Figure 1:**
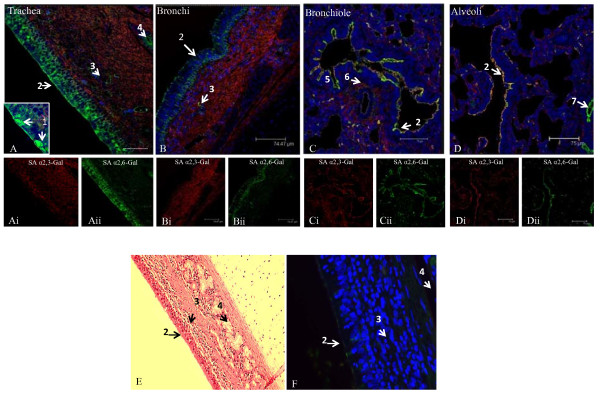
**Differential expression of SAα2,6-Gal (SNA lectin) and SAα2,3-Gal (MAA II lectin) receptors in the porcine respiratory tract**. Composite confocal images show distribution of SAα2,6-Gal receptors (green) and SAα2,3-Gal receptors (red) with nuclear staining (blue). Representative results that show SAα2,6-Gal receptor as the dominant receptor type on the epithelium of trachea (A), bronchus (B) and bronchiole (C), where epithelial cells and goblet cells are the main contributing cell types. SAα2,3-Gal receptor, on the other hand, is the major receptor in the corresponding sub-epithelial (mucosal) region with sparse concentration of SAα2,6-Gal receptor at blood vessels and mucous/serous glands. Both receptor types are similarly expressed on alveolar lining (D). The specificity of lectin binding is demonstrated on serial tracheal sections stained with haematoxylin and eosin (E), and with both SNA and MAA II lectins on section previously treated with sialidase A, where only faint background binding is detected (F). 1. goblet cell, 2. epithelial lining, 3. gland with occasional blood vessel, 4. submucosal gland, 5. mucosa, 6. smooth muscle, 7. blood vessel. Scale bar = 75 μm.

Along the upper respiratory lining of trachea and bronchus, SA α2,6-Gal receptor (fluorescein isothiocyanate [FITC] labelled SNA) was dominant in the ciliated pseudostratified epithelia, in which were found mucus secreting goblet cells (Figure 1A, B, C, E and 1F). The relative abundance of SA α2,6-Gal receptor along the large airways continued down to the cuboidal epithelia of bronchioles. Additionally, there was a gradual relative rise in expression of SA α2,3-Gal receptor (biotinylated MAA II) towards the lower respiratory lining, such that in the alveolar lining both SA α2,6-Gal and SA α2,3-Gal receptors were similarly expressed and with a degree of co-localisation of expression (Figure 1D).

**Figure 2:**
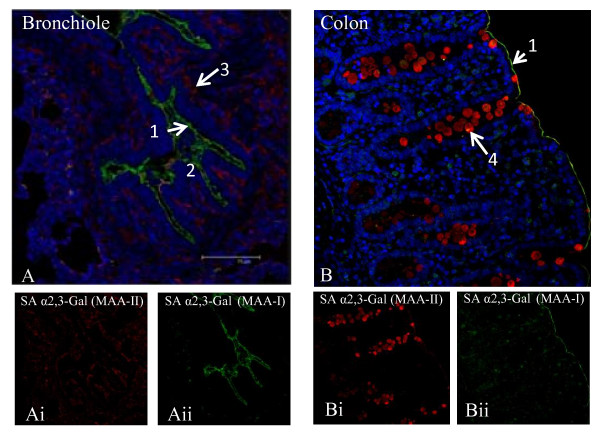
**Discriminating two types of SA α2,3-Gal receptors in bronchiole (A) and colon (B) by FITC labelled MAA I (green), and biotinylated MAA II (red) receptors**. In bronchiole, MAA I and MAA II typically show similar binding intensity, with prominent presence of MAA I on the epithelial lining (Aii). In colon, MAA II binding, mainly localised to goblet cells, dominates MAA I; MAA I binding is seen as a fine line bordering the epithelium (Bii). 1. epithelial lining, 2. mucosa, 3. smooth muscle, 4. goblet cell. Scale bar = 75 μm.

Interestingly, in the lamina propria (mucosa) of the respiratory tract, SA α2,3-Gal (MAA II) was dominant over SA α2,6-Gal receptor (Figure 1A, B and 1C). At these sub-epithelial locations, the less abundant SA α2,6-Gal receptor was mainly confined to mucous/serous glands. To further discriminate between SA α2,3-Gal receptor subtypes, MAA I lectin (SAα2,3-Galβ(1-4)GlcNAc specific) was used in comparison with the more commonly used MAA II lectin (SAα2,3-Galβ(1-3)GalNAc detection). MAA I receptor subtype was not detected in trachea and bronchus (data not shown). However, MAA I receptor was relatively more highly expressed than MAA II at the epithelial lining of the lower respiratory tract (bronchioles and alveoli) (Figure 2A).

### SA receptors distribution along intestinal tract

**Figure 3:**
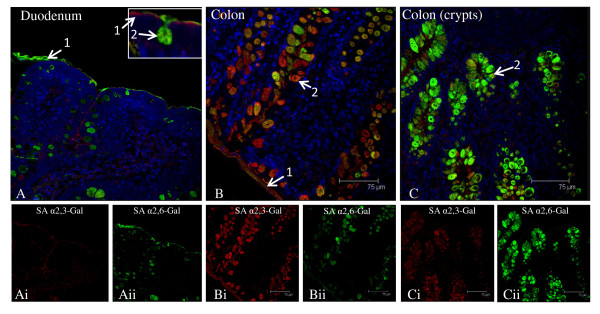
**Differential expression of SAα2,6-Gal (SNA lectin) and SAα2,3-Gal (MAA II lectin) receptors in the porcine intestinal tract**. Composite confocal images show distribution of SAα2,6-Gal receptors (green) and SAα2,3-Gal receptors (red) with nuclear staining (blue). Representative results that show the spatial distribution of both receptor types in the duodenum (A) and colon (B, C). In duodenum, SAα2,6-Gal receptor is more abundant than SAα2,3-Gal receptor concentrated in goblet cells and along the epithelial lining (A). In colon, strong co-expression of SAα2,6-Gal and SAα2,3-Gal receptors is detected in goblet cells and on epithelial lining. Colon goblet cells at the crypts show a higher concentration of SAα2,6-Gal receptor (Cii) than those located towards the luminal surface (Bii). 1. epithelial lining, 2. goblet cell. Scale bar = 75 μm.

In the duodenum, both SA α2,3-Gal and SA α2,6-Gal receptor types were detected (Figure 3A). However, SA α2,3-Gal receptor (MAA II detection) was weakly expressed, mainly confined to parts of the epithelial border. SA α2,6-Gal receptor (SNA positive) was dominant and localised along the epithelial border and in goblet cells. There was progressive increase of SA receptors towards the lower gut (Figure 3B and 3C). In the colon, both SA α2,3-Gal and SA α2,6-Gal receptors were strongly detected on the epithelial border and in goblet cells. Co-expression of the two receptors in goblet cells was also frequently observed. Furthermore, there appeared to be an expression gradient of SA α2,6-Gal receptor along the column of goblet cells in the colon; more SA α2,6-Gal receptor was found in the crypt region than towards the luminal surface (Figure 3Cii and 3Bii). To further discriminate between SA α2,3-Gal receptor subtypes, MAA I lectin (SAα2,3-Galβ(1-4)GlcNAc specific) was used in comparison with MAA II lectin (SAα2,3-Galβ(1-3)GalNAc) binding. There was no MAA I lectin binding in the duodenum (data not shown). The weak presence MAA I was mainly confined to a thin border along the colon epithelium (Figure 2B).

### SA receptors in other major organs

**Figure 4:**
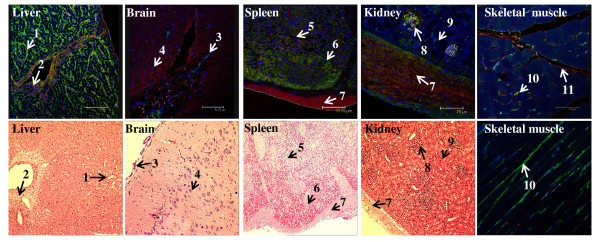
**Extensive presence of SAα2,6-Gal (SNA lectin) and SAα2,3-Gal (MAA II lectin) receptors in the major porcine organs examined**. Composite confocal images, along with corresponding haematoxylin and eosin tissue sections (with the exception of skeletal muscle) for orientation, show distribution of SAα2,6-Gal receptors (green) and SAα2,3-Gal receptors (red) with nuclear staining (blue). Cross section and longitudinal section of skeletal muscle are shown. 1. hepatic sinusoid, 2. portal triad, 3. meninx, 4. neuron, 5. white pulp, 6. red pulp, 7. capsule, 8. glomerulus, 9. renal tubule, 10. capillary, 11. basement membrane.

In the liver, SA α2,6-Gal receptor was most prominent along the sinusoid-hepatocyte boundary, suggesting that sinusoidal endothelial cells and/or Kupffer cells could be the principal cell type(s) for SA α2,6-Gal receptor expression (Figure 4). SA α2,3-Gal receptor (MAA II specific) in the liver was mainly distributed in the connective tissue, such as around the portal triad region. In the brain, neuronal cells predominantly expressed SA α2,3-Gal receptor, with sparse presence of SA α2,6-Gal receptor that appeared to localise to meningeal blood vessels, presumably endothelial cells (Figure 4). In the spleen, both receptor types were diffusely expressed throughout the organ, with concentration of SA α2,6-Gal receptor in the pulp areas which are rich in lymphocytes (Figure 4). In the kidney, both SA α2,3 and SA α2,6-Gal receptors were largely restricted to glomeruli, with the occasional SA α2,6-Gal positive tubular cells. The renal capsule as well as splenic capsule were sites of mainly SA α2,3-Gal expression. In skeletal muscle, SA α2,6-Gal receptor appeared to be confined to blood capillaries and SA α2,3-Gal receptor was detected along the basement membrane of muscle fibre (Figure 4).

### Correlation between influenza virus subtype and SA receptor type

**Figure 5:**
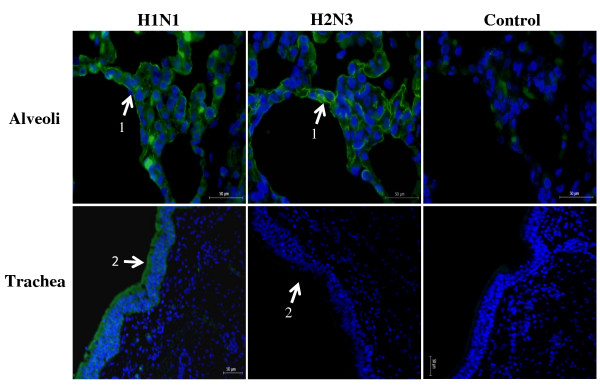
**Virus binding assays with swine H1N1 and avian H2N3 viruses on porcine alveolar and tracheal serial sections are consistent with virus affinity for particular host receptor type**. The presence of both SAα2,6-Gal and SAα2,3-Gal receptors in alveoli is mirrored by a similar overlapping binding pattern of swine and avian viruses to pneumocytes. By contrast, the dominant SAα(2,6)-Gal receptor type on tracheal epithelium shows preferential binding of swine H1N1 virus. Control sections are without virus treatment; low level of auto-fluorescence was detected on alveolar section. 1. alveolar pneumocyte, 2. epithelial lining. Scale bar = 50 μm.

To establish a functional correlation between SA receptor types and binding affinity of influenza virus subtypes (mammalian and avian), virus binding assays were performed on lung and tracheal tissue sections (Figure 5). As predicted, avian H2N3 and swine H1N1 (human-like) viruses bound to lung alveoli with similar affinity and with overlapping spatial distribution, consistent with the spatial arrangement of the two main SA receptor types. On tracheal sections, only swine H1N1 strongly bound along the epithelial border and specifically, but less strongly, to the ciliated pseudostratified epithelium. Avian H2N3 virus showed little or no binding affinity for tracheal epithelium as was predicted.

## Discussion

**Figure 6:**
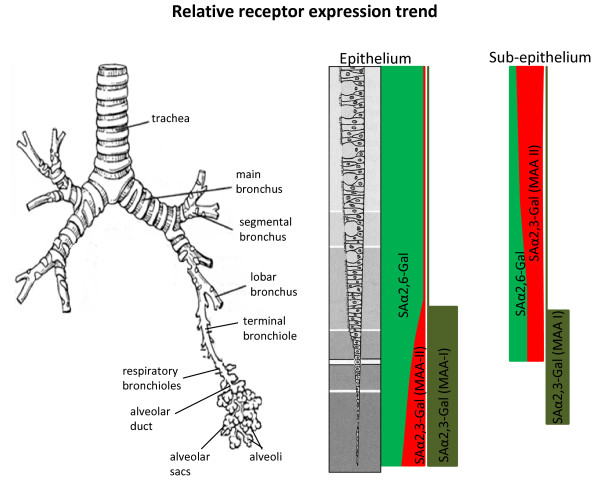
**Schematic representation of the distribution trend of SAα2,6-Gal (SNA), SAα2,3-Galβ(1-4)GlcNAc (MAA I) and SAα2,3-Galβ(1-3)GalNAc (MAA II) receptors along the porcine respiratory tract**. Diagram depicts a qualitative, not quantitative, assessment of receptor presence. Along the epithelial tract, SAα2,6-Gal receptor is dominant, with increasing MAA II lectin binding towards the alveolar region. In the sub-epithelial region, MAA II lectin binding is dominant. MAA I lectin binding is localised to the lower tract.

To date, there has been no detailed account published on the distribution of SA receptors in the pig, an important mammalian host for influenza infections [14,15]. In this study, we found extensive presence of SA α2,6-Gal (human) and SA α2,3-Gal (avian) receptors in the major organs examined which suggests that each organ is potentially a target for influenza virus entry or infection. In the porcine respiratory tract, SA α2,6-Gal receptor was dominant in the epithelia of the upper tract (trachea and bronchus) (Figure 1). As the whole of the upper respiratory tract are architecturally identical, characterised by ciliated pseudostratified squamous epithelia interspersed with goblet cells and subepithelial structures of mucous/serous glands, we expect lectin binding patterns in the nasal turbinate and larynx to be highly similar to the reported trachea and bronchus. Towards the lower epithelial tract (bronchiole and alveolar region), there was relative increase of SA α2,3-Gal (MAA II) receptor, along with the continued presence of SA α2,6-Gal receptor (Figure 6). By contrast, SA α2,3-Gal receptor distribution (detected by MAA II and I lectins) was widespread in the sub-epithelial mucosa of the respiratory tract (Figure 1 and 6), which suggests that the respiratory mucosa is potentially permissive to avian influenza virus replication if the epithelial lining is breached. Our tracheal findings are broadly in agreement with a previous observation that avian and human receptors are located in porcine tracheal epithelium [10]. It should be pointed out that the source of MAA lectin (SA α2,3-Gal detection) used in that study and the likelihood of pig variation could account for its more intense detection signal compared with our use of a more specific MAA II lectin [16].

Our findings regarding SA receptor distribution along the porcine respiratory are similar to reported human data (Figure 4). In human trachea, SA α2,6-Gal receptor was abundantly expressed on ciliated and non-ciliated epithelial cells and SA α2,3-Gal receptor was only sparsely detected (on non-ciliated cells) [17]. On human non-ciliated cuboidal bronchiolar epithelial cells SA α2,3-Gal receptor was readily detected [18]. In alveoli, SA α2,6-Gal receptor showed diffused expression in different cell types (type I and II pneumocytes and alveolar macrophages) whereas the greater prominence of SA α2,3-Gal receptor was restricted to type II pneumocytes [17,18]. Although in our pig study, no specific staining was made to distinguish the different cell types along the respiratory epithelia, the relative expression of SA α2,6-Gal and SA α2,3-Gal (MAA II) receptors from trachea to alveolar region is a close match to the human pattern of expression. Indeed, recent improvements in lectin-binding specificity and sensitivity have shown that SA α2,6-Gal as well as SA α2,3-Gal receptors are often both found in different cells types (ciliated epithelial, goblet and submucous gland), unlike previous reports that indicated certain type of cells had only one lectin-binding profile [16].

To further discriminate SA α2,3-Gal receptor binding, MAA I and MAA II lectins were used. In porcine trachea and bronchus, MAA I binding was undetectable. However, on the epithelial lining of porcine bronchiole and alveoli, MAA I was more dominant than MAA II (Figure 2Aii and 6). The differential distribution of MAA I and MAA II in pig appears to be different from human. In human, MAA I shows widespread binding throughout the upper and lower respiratory tract, and MAA II binding was mainly restricted to the alveolar epithelial cells of the lung [16]. It has been suggested that in human cases of highly pathogenic avian H5N1 infection, the entry of H5N1 virus in the upper respiratory tract is mediated by MAA I lectin specific receptors [16]. As domestic pigs are inherently resistant to H5N1 infection [11], it is interesting to speculate that such resistance could be connected to the relative absence of MAA I specific receptor on the porcine upper respiratory tract (Figure 4 and 6).

On porcine epithelia of small and large intestines, both SA α2,6-Gal and SA α2,3-Gal (MAA II lectin) receptors were clearly detected in goblet cells, as the main SA receptor cell type (Figure 3). There was more MAA II binding in the colon than duodenum. We further reported the presence of MAA I binding in the colon but not in the duodenum (Figure 2B). It is not apparent why goblet cells located in the colon crypts showed a greater abundance of SA α2,6-Gal expression than those in the apical region. The little available data on the characterisation of human SA receptors in the intestinal tract showed the absence of both SA α2,6-Gal and SA α2,3-Gal receptors in small and large intestines [17] or absence of SA α2,3-Gal receptor in colon epithelium [19]. This is surprising given the abundance of mucin-secreting cells in the intestinal tract especially in colon epithelium. The reported lack or absence of SA receptors in human intestinal epithelia could be attributed to the preservation state of human gut samples and/or the use of less sensitive detection techniques for lectin binding. Given that diarrhoea is not an infrequent presenting sign of influenza infection in mammals, such as avian H5N1 infection in humans [20] and the 2009 pandemic A/H1N1 infection in pigs [21], there is a need for clarification regarding SA receptor distribution in the human gut.

The spatial distribution of SA α2,6-Gal and SA α2,3-Gal (MAA II) receptors in porcine liver, brain, spleen, kidney and skeletal muscle closely resembled the distribution in human organs (Figure 4) [17]. In human liver, SA α2,6-Gal receptors are found on hepatocytes and Kupffer cells. In human brain, neuronal cells show abundance of SA α2,3-Gal receptor. In human spleen, both receptor types are localised to T- and B-lymphocytes (pulp areas). In human kidney, glomeruli are the foci of SA α2,6-Gal and SA α2,3-Gal expression. In human cardiac muscle, SA α2,6-Gal receptor is detected in endothelial cells [17]. These comparative observations suggest that the major organs examined in the pig, as in humans, are potentially susceptible to the viraemic spread of influenza virus, and that the brain could be particularly susceptible to virus encephalitis from an avian subtype [22]. In experimental pigs intra-nasally infected with a low pathogenic avian H5N2 subtype, virus recovery was made from the brain stem of 3 out of 12 animals which highlights neural tissues as potentially susceptible to avian influenza virus infection [23]. Based on the extensive similarity in the expression and distribution of SA α2,6-Gal and SA α2,3-Gal (MAA II) receptors between pig and human in the major organs, in particular, in the respiratory tract (with the exception of inadequate receptor data on human intestines) it is suggested that the pigs are not more likely to act as "mixing vessels" for influenza virus reassortment between avian and mammalian subtypes than humans.

## Conclusion

We established that both human influenza (SA α2,6-Gal) and avian (SA α2,3-Gal, MAA II specific) receptor types are extensively present in all pig organs examined, with each tissue showing distinctive spatial distribution of the two receptors. This suggests that each major organ may be permissive to virus entry or infection. Based on SA distribution similarity, pigs appear not more likely to be potential hosts for virus reassortment than humans. The relative abundance of SA receptors in the pig intestines highlights a need for clarification on the presence of SA receptors and their potential significance in the human intestinal tract.

## Methods

### Pig tissues

Four 4 to 8 week-old healthy post-weaned male commercial pigs, sourced locally, were euthanized in adherence to Home Office regulations from which trachea, lung, liver, kidney, spleen, heart, skeletal muscle, cerebrum, small intestine and colon samples were taken and fixed in 10% buffered neutral formalin. Serial sections of paraffin embedded tissue slides (5 μm thickness) were generated for histological analysis.

### Lectin histochemistry

Detection details of host influenza receptors are found in Kuchipudi et al. (2009) [24]. Briefly, sections were pre-soaked in Tris-buffered saline (TBS) and blocked using a biotin-streptavidin blocking kit (Vector Laboratories) according to manufacturer's instructions, followed by overnight incubation at 4°C with FITC labelled SNA or FITC labelled MAA I, and biotinylated MAA II lectin, each at a concentration of 10 μg/ml. After three washes with TBS, the sections were incubated with streptavidin-Alexa-Fluor594 conjugate (Invitrogen) for 2 h at room temperature (RT). The sections were washed and mounted with ProLong Gold anti-fade reagent with 4', 6-diamino-2- phenylindole, dihydrochloride (DAPI; Invitrogen). Negative controls were performed omitting the primary reagents. To rule out non-specific binding of the lectins, tissue sections were treated, prior to lectin staining, with sialidase A (N-acetylneuraminate glycohydrolase; Prozyme) for 24 h at 37°C (pH 6.0), which preferentially cleaves all non-reducing terminal sialic acid residues in the order α(2,6) > α(2,3) > α(2,8) > α(2,9). Images were captured by confocal microscopy (Leica TCS SP2 AOBS).

### Virus binding to host tissues

Host receptor binding assays with H1N1 classical swine strain (A/Sw/Iowa/15/30), a subtype closely related to the human 1918 pandemic influenza virus [25], and an H2N3 low pathogenic avian strain (A/mallard duck/England/7277/06) were performed as previously described, with minor modifications [26]. Briefly, paraffin embedded 5 μm sections of lung tissues were deparaffinised in xylene and rehydrated by alcohol. Deparaffinised tissue sections were incubated with TPCK trypsin treated avian or swine influenza virus for 24 h at 37°C. Paradoxically, we found that mammalian H1N1 virus binds more efficiently at 37°C than at the usual 4°C. The sections were washed, blocked with goat serum for 30 min, and incubated with a mouse monoclonal antibody specific for influenza nucleoprotein (Abcam, Cambridge, UK) in 1:1000 dilution, overnight in a humidified chamber at 4°C. A secondary antibody, FITC-labelled goat anti-mouse IgG (Abcam, Cambridge, UK), was applied at 1:500 dilution for 2 h at RT. After three further washes with TBS, the sections were mounted with ProLong Gold anti-fade reagent with DAPI.

## Authors' contributions

RKN performed most of the experimental work and contributed to the writing process. SVK, GAW, BBP and SPD assisted with experimental work and provided intellectual input to the work. KCC initiated, coordinated and wrote the manuscript. All authors read and approved the final manuscript.
